# Complementary support in later life: investigating the gender disparities in patterns and determinants among older adults in South-Western Nigeria

**DOI:** 10.1186/s12877-022-03393-w

**Published:** 2022-08-25

**Authors:** Jacob Wale Mobolaji, Akanni Ibukun Akinyemi

**Affiliations:** Department of Demography and Social Statistics, Obafemi Awolowo University, Ile-Ife, Nigeria

**Keywords:** Complementary support, Social support, Social network, Support sources, Older adults, Nigeria, Sub-Saharan Africa

## Abstract

Old-age needs are multifaceted and require multiple support sources, yet caregiving roles for older Nigerians are largely shifted to adult children. However, the children also declining capacity to respond. The extent to which older adults access support from other sources remains under-researched. This study investigates the patterns and determinants of access to complementary supports among older adults in South-Western Nigeria, taking Oyo State as the case study. The study is cross-sectional and utilized primary data of 827 older adults aged ≥ 65 years selected using a multi-stage sampling design. Box plot was used to determine the patterns while multiple ordinary least square regression was used to predict the determinants of access to complementary support. Expressed in percentage, the median complementary support score of older adults in Oyo State was 30 (interquartile range [IQR] = 24) with a slightly higher score for men (median = 32, IQR = 24) compared to women (median = 28, IQR = 20). Access to complementary support was lower for the widow(er)s, the lower socioeconomic group and self-dependent older adults across genders, and for urban women with secondary/higher education compared to the otherwise groups. Increased access to complementary support was significantly associated with primary/no education (β = 4.365; *p* < 0.01 95% C.I. = 1.511–7.218), affiliation to Islamic/Traditional religion (β = 5.100; *p* < 0.001; 95% C.I. = 3.000–7.200), rich wealth status (β = 3.315; *p* < 0.05; 95% C.I. = 0.667–5.963) and depending on both self and children/family for income (β = 5.510; *p* < 0.05; 95% C.I. = 1.710–9.309) with some gender disparities. However, reduced complementary support was associated with ages 80 years or over (β = -3.649; *p* < 0.05; 95% C.I. = -6.460 – -0.838) and widowhood (β = -6.285; *p* < 0.001; 95% C.I. = -8.556 – -4.015). The study suggests the need for welfare plans among professional, social, and religious groups, institutionalised social support systems, and community engagement to escalate welfare support for older adults. It also recommends intensified attention on the more vulnerable groups, especially the widows, childless and lower socioeconomic groups.

## Introduction

Later life is associated with an increased need for assistance to accomplish usual tasks [1, 2] due to social, economic, and functional changes. The period is usually marked by disengagement from the labour force, particularly for those in formal employment, which further reduces their social engagement chances. Those in the informal setting are less agile to work and earn income that is sufficient to cater to their needs, while those at much-advanced age may become functionally limited in carrying out their activities of daily living including personal care, dressing, bathing, shopping, preparing a meal, getting water, mobility among others. Others may struggle with loneliness, social isolation, and depression [3–6]. These conditions in later life necessitate dependence on support for survival.

However, supports are largely limited to informal sources in many sub-Saharan African (SSA) societies like Southwestern Nigeria, with poor or non-functional social welfare systems for the aged. The informal supports comprise those from family, friends, neighbours and social groups, and community members. The traditional family support networks, the major sources of support for older adults are growing weak in Southwestern Nigeria [7, 8]. This is because the African traditional family constellations are rapidly being replaced with modernity [9] which emphasises the nuclear family and shifts caregiving roles to adult children of the older adults. This is especially the case in Southwestern Nigeria where children have limited capacity to provide adequate care for aged parents due to the social changes, migration, and economic downturn [10]. This turn of events points to the increasing need for alternative sources of support to complement the weakening sources.

Complementary support from multiple sources is imperative for achieving optimum well-being in later life, amidst the declining support from immediate family. Complementary support, first used by Litwak (1985), is a model which postulated that formal support serves as a fall-back to complement the failure of other support sources [11]. This model is guided by the assumption that supports from the government and other formal organizations exist to complement informal support from immediate families and non-family social networks. Nevertheless, the principle of complementary support is also applicable to informal support sources such that support from non-family members complements the support from the primary caregivers, usually children and spouse or some kin where children and spouse do not exist.

The concept of complementary support presents an opportunity for older adults to get additional support from a spouse, children, relatives, and non-relatives, especially when it is lacking or insufficiently received from certain primary sources [11, 12]. Some older adults combine support from family, friends, and church members when necessary [13, 14]. However, the extent of this opportunity varies, depending on the availability and viability of the support networks. Access to support from multiple sources serves as a buffer for an older adult, in case of loss of any sources of support due to either death or relationship breakdown [15].

Individuals without a spouse and/or children are at risk of support deprivations from those sources. This, particularly, implies that the unmarried and the childless could be limited to receiving support from distant relations and non-relatives who may not oblige to help [16, 17]. This evidence echoes the importance of complementary support. Thus, building a wide social network with positive relationships is key to expanding access to multiple support in later life.

Meanwhile, the extent of access to complementary support may be influenced by some factors including the recipients’ characteristics and types of relationships. For instance, a few studies have associated access to support from the social networks with having a large social network [18] and closeness to the network members [19]. Others have associated access to social support with age, level of education, and marital status [4, 20–24]. These studies, however, are largely in the context of high-income countries. Such evidence is lacking in Nigeria, a country with the highest population of older adults in Africa and an estimated 90 million of her population living in poverty [25].

In addition, gender inequalities often exist in access to support by older people. Older men tend to suffer more neglect, hence more unmet needs for support, than women. For instance, evidence suggests that aged mothers tend to receive more support than fathers from children, especially daughters [26, 27]. This mother-children relationship is common in many cultures, including the Yoruba culture of Southwestern Nigeria. Greater support in favour of women compared to men might be linked to women’s tendency to live more closely with children, unlike men. Conversely, older husbands living with younger and healthy wives benefit more from spousal support than older married women [27, 28]. However, due to the pervasive traditional belief system in Southwestern Nigeria, some aged women are liable to being neglected, stigmatised, and labelled as witches and occult individuals. These gender disparities result in greater unmet needs, poorer health, and lower life expectancy for the less-advantaged gender.

Gender disparities in access to complementary support have been given less attention in the study of aging in sub-Saharan Africa (SSA), particularly in Nigeria. This study aims at investigating the access to complementary support and the associated factors to examine the gender variations among older adults in Southwestern Nigeria. To the best of our knowledge, this is the first study assessing older adults’ access to complementary support in Nigeria; and of the existing gerontological studies in the country, none has investigated the associated factors of social support from the point of view of complementary support.

## Methods

### Study design and data sources

The study focuses on older adults aged ≥ 65 years. Though there are alternative definitions, for this study, we chose the local retirement age of 65 years as a cut-off point. The study was based on quantitative primary data collected from older adults age ≥ 65 years in Oyo State, the second largest of the six states in Southwestern Nigeria. The state with an estimated total population of 6,728,829 and 482,747 older adults of ages 60 or older has the second-highest population of older adults in Southwestern Nigeria after Lagos. The State has a higher proportion of older adults among the male population (8.1%) compared to the female population (4.2%), unlike the situation in other Southwestern states [29]. This disparity prompted the selection of the state for this study. Though the reason for the disparity remains unclear and requires empirical evidence, it suggests a lower survival rate among older women compared to men, higher female births than mal, es or increased mortality of male children.

The samples for this study were selected using a multistage cluster sampling procedure. Oyo State has three senatorial districts – Oyo North, Oyo South, and Oyo Central, each comprising 11, 13, and 9 local government areas (LGAs) respectively. Two LGAs were randomly selected from each senatorial district: Atisbo and Ogbomoso South LGAs from Oyo North; Ibarapa East and Ibadan North-West LGAs from Oyo Central, and Afijio and Lagelu LGAs from Oyo South senatorial district. A total of 30 clusters, also referred to as the primary sampling unit (PSU) were selected from all the LGAs using a systematic random sampling technique. Oyo State enumeration areas (EAs) developed for the 2006 population census frames were used as the sampling frame for the selection of the clusters, each cluster containing three or four EAs. A total of 111 EAs from which households with eligible respondents were selected for the study. All persons, either male or female, aged ≥ 65 years in the selected households were interviewed, excluding those with dementia or chronic sickness.

The study was based on a total sample size of 827 older adults, 394 men and 433 women, aged ≥ 65 years. The sample size was determined based on the population of older men and women and the prevalence of unmet needs among them. In this regard, the sample size for comparing two proportions was used. For each aged men and women population, a sufficient and proportional sample size *n* was calculated using the approach recommended by Casagrande, Pike, and Smith [30] and Fleiss, Tytun, and Ury [31] as follows:

$$n = \frac{{n}^{^{\prime}}}{4}{\left[[1+\sqrt{1+\frac{2\left(r+1\right)}{r{n}^{^{\prime}}\delta }}]\right]}^{2}$$

where $${n}^{^{\prime}}=\frac{{\left[{Z}_{\alpha }\sqrt{\left(r+1\right)\overline{P} }\,\overline{Q }+{Z}_{\beta }\sqrt{(r{P}_{1}{Q}_{1}+{P}_{2}{Q}_{2})}\right]}^{2}}{r{\delta }^{2}}$$

$$\delta=\left|P_2-P_1\right|;\overline P=\frac{P_1+rP_2}{r+1};\overline Q=1-\overline P$$

$$N={(r+1)}^\ast n$$

From a previous study, the proportion of older women (P_1_) and men (P_2_) with unmet needs for informal support in Southwestern Nigeria was estimated as 0.491 and 0.616 respectively [10]. This study utilised the prevalence of the unmet need for informal support to determine the sample size in the study location. The sample size was based on a 5% level of significance (α) and 10% precision power (β), and a sex ratio of 0.91 males per female of Nigerian older adults aged ≥ 65 years [32]. The sample selection was distributed based on the rural–urban strata and the LGAs selected from each Senatorial District.

The data were collected by 10 research assistants, largely postgraduate students who were recruited and trained on electronic data collection using Android devices, and three field supervisors who supervised the exercise to ensure quality data. The data which were collected over 11 days were monitored and checked in real-time daily for any irregularity and incomplete data, for immediate correction by the research assistant concerned. The data relating to the respondents’ sociodemographic characteristics, social support networks, types, and extent of support received were collected and managed electronically using Research Electronic Data Capture (REDCap) tools hosted at the University of the Witwatersrand, Johannesburg, South Africa [33, 34]. The REDCap, as a secured web-based software was used to capture and securely store the research data before being exported to Stata 15.1 statistical programme.

### Variables and measures

The outcome variable for this study is the extent of access to complementary support by older adults. It was a composite score computed based on the extent of support received from the various sources. Fifteen support sources were covered: 2 sources (children, spouse) grouped as immediate families, 3 sources (siblings, kin, and in-laws) as extended families, and 10 sources (neighbours, friends, religious groups, social groups, community members, professional groups, government, politicians, philanthropists, and NGOs) as non-relatives. The community members in this study refer to the members of the community of residents who are neither the friends nor direct neighbours of the respondents but have contact with them. The social group refers to non-religious and non-professional social organisations the older adults belong to including social or sport clubs, self-help groups, cooperative societies, and class/schoolmate associations whose members are not necessarily residents in the respondents’ communities but are connected.

For each of the 15 support sources, the respondents were asked about the extent to which they received support for their needs in the last 12 months. The questions imply the respondent’s receipt of multiple and/or frequent support from the support sources within the reference period. Existing studies have recalled a 12-month past event from older adults and have reported no recall bias [35]. A 12-month period was chosen to capture less frequent episodes of support, for example, receiving material or health care provision. The response for each source of support was scored on a 5-point Likert scale: 1 for never received any support, 2 for receipt of very little support, 3 for receipt of moderate support, 4 for receipt of much support, and 5 for receipt of lots of supports. The scores for the 15 support sources were summed up to obtain the total complementary support score of each respondent out of the total possible score of 75. However, for ease of interpretation, the scores were expressed in percentage. The reliability of the scale was tested using the Cronbach Alpha statistic (α = 0.842), which indicates that the scale is very reliable.

The explanatory variables for this study include the age, grouped into below 70, 70–74, 75–79, 80–84 years, and 85 years or older; level of education – categorized as none, primary, secondary/higher education; marital status categorized as single, married and living with a partner, separated, divorced and widowed; religion categorized as Christianity, Islam and Traditional; sex as male and female; rural–urban place of residence; and wealth status categorized as low, middle and high. The wealth status was generated using a composite score of the household facilities possessed by the respondents: telephone, television, radio, fan, refrigerator, motorcycle, car, modern toilet facility, house wall, roof and floor condition, and presence of electricity. The composite score was divided into tertile. Other explanatory variables are economic activity status coded as working and not working and main sources of income, categorised as self only, family only, and both self/family.

### Statistical analysis

The data were analysed using various statistical data presentation approaches. Percentage distribution was used to present the socio-demographic characteristics of the respondents. Box plots were used to depict the gender disparities and patterns of access to complementary support by the study participants. At the multivariate level, having established the basic assumptions of linearity and normality (skewness < 0.000, Kurtosis = 0.2159) test of the complementary support score, multiple linear regression analysis was used to identify the socio-demographic factors associated with the extent of access to complementary support among the older adults in the study area. The regression models follow the regression equation below:

$$\widehat{\mathrm Y}=b_0+b_i^\ast X_i+\dots+b_n^\ast X_n+e_i$$

where *Ŷ* is the estimated value of the outcome variable; *b*_*0*_ is the Y-intercept or the value of the outcome variable *Y* when the explanatory variables *X*_*i*_ = 0; *b*_*i*_ is the regression coefficient or magnitude of changes in Y for a unit change in each of the explanatory variables *X*_*i*_*, i* = *1, 2, 3,…, n* while *e*_*i*_ is the residual error term, also regarded as the unexplained error (i.e. the average squared difference between the predicted and the observed values). All the multivariable analyses were based on a 95% confidence interval for the complementary support score.

In this study, the complementary support score generated as described above is the outcome variable Y_*i*_ while age, place of residence, level of education, marital status, religion, wealth status, occupational status, and main sources of income are the explanatory variables *X*_*1*_* to X*_*n*_ each of which was converted to dummy variables to estimate the outcome variable as follows.

*Extent of complementary support* = *b*_*0*_ + *b*_1_*(age 70–79) + *b*_2_*(age ≥ *80)* + *b*_3_*(urban) + *b*_4_*(primary/no education) + *b*_5_*(never married/separated/divorced) + *b*_6_*(widow) + *b*_7_*(other religions) + *b*_8_*(middle wealth status) + *b*_9_*(high wealth status) + *b*_10_*(working) + *b*_11_*(income from children/family) + *b*_12_*(income from self and children/family) + *e*_*i*_*.*

The otherwise category in each of the explanatory variables was the reference category (code = 0). The regression model above was estimated for male, female and overall data using the forced entry linear regression method.

## Results

### Sociodemographic characteristics of the respondents

Table 1 shows the socio-demographic distribution of the respondents in this study. According to the table, about two-thirds were aged 70 years or older, engaged in economic activities, and over three-fifths in Christian religion for both genders. A higher proportion of men 277 (70%) were currently married compared to their women counterparts 127 (29%); while a larger proportion of the women 283 (65%) were widows compared to men 69 (18%). The proportion with secondary/higher education was higher among men 97 (25%) compared to women 43 (10%). About six in 10 of the older adults, irrespective of gender, resided in an urban area; and were self-reliant for income. A higher proportion of men 260 (66%) compared to women 233 (52%) reported that they relied on their income, while more women depended on children/family for income 177 (41%) compared to men 97 (25%).

**Table 1:** Background characteristics of older adults in Oyo State, Nigeria

Socio-demographic characteristics	Male N_m_ = 394	Female N_f_ = 433	Overall *N* = 827
	n (%)	n (%)	n (%)
Age group			
65 – 69	131 (33.2)	147 (34.0)	278 (33.6)
70 – 74	90 (22.8)	89 (20.6)	179 (21.6)
75 – 79	70 (17.7)	57 (13.2)	127 (15.4)
80 – 84	54 (13.7)	74 (17.1)	128 (15.5)
85 +	50 (12.6)	65 (15.1)	115 (13.9)
Level of education			
None	129 (32.7)	272 (62.8)	401 (48.5)
Primary	168 (42.6)	118 (27.2)	286 (34.6)
Secondary/equivalent	63 (16.0)	31 (7.2)	94 (11.4)
Tertiary	34 (8.6)	12 (2.8)	46 (5.5)
Marital Status			
Never married	4 (1.0)	0 (0.0)	4 (0.5)
Married, living with a spouse	277 (70.3)	127 (29.3)	404 (48.8)
Separated	36 (9.2)	21 (4.8)	57 (6.9)
Divorced	8 (2.0)	2 (0.5)	10 (1.2)
Widowed	69 (17.5)	283 (65.4)	352 (42.6)
Place of residence			
Rural	165 (41.9)	191 (44.1)	356 (43.1)
Urban	229 (58.1)	242 (55.9)	471 (56.9)
Religion			
Christianity	238 (60.4)	278 (64.2)	516 (62.4)
Islam	149 (37.8)	303 (36.6)	303 (36.6)
Traditional	7 (1.8)	1 (0.2)	8 (1.0)
Wealth status			
Poor	90 (22.8)	134 (31.0)	224 (27.1)
Middle	111 (28.2)	83 (19.2)	194 (23.5)
Rich	193 (49.0)	216 (49.9)	409 (49.5)
Occupational Status			
Not working	132 (33.5)	152 (35.1)	284(34.3)
Working	262 (66.5)	281 (64.9)	543 (65.7)
Main sources of income			
Self (work/pension)	260 (66.0)	223 (51.5)	483 (58.4)
Children/family	97 (24.6)	177 (40.9)	274 (33.1)
Both self and children/family	37 (0.4)	33 (7.6)	70 (8.5)

### Available sources of support for older adults in Oyo State

The results in Table 2 indicate the proportion of older adults in Oyo State receiving substantial (much or a lot of) support from the available social networks in the past 12 months. Overall, the most common sources of support were the children, with over 85% of older adults reporting receipt of substantial support from the children; however, a slightly higher proportion of women 356 (88%) compared to men 308 (82%) enjoyed children support. The next important source of support was the spouse with about 340 (76%) reporting access to substantial support from the spouse. Only about half 407 (49%) of the older adults could boast of substantial support from siblings, and 276 (33%) from other relatives/kin and In-laws each. About 2 or 3 in 10 older adults received appreciable support from neighbours, friends, and religious groups, while less than 10% accessed support from each of the other sources.

**Table 2:** Proportion of Older Adults Receiving Significant Support from Social Networks in Oyo State

Support sources	Male *n* = 394	Female *n* = 433	Unmarried ^C^ *n* = 431	Childless *n* = 33	Overall *N* = 827
*Immediate family*					
Children ^b^	308 (82.1)	356 (87.7)	354 (82.2)	-	664 (85.0)
Spouse ^a^	230 (75.2)	110 (76.4)	-	7 (21.3)	340 (75.6)
*Extended family*					
Siblings	193 (49.0)	215 (49.7)	202 (46.9)	17 (51.5)	407 (49.3)
Other relatives	131 (33.3)	145 (33.5)	145 (33.7)	17 (51.5)	276 (33.4)
In-laws	132 (33.5)	141 (32.8)	147 (33.9)	11 (33.3)	273 (33.0)
*Non-family members*					
Neighbours	113 (28.6)	122 (28.2)	127 (29.5)	11 (33.3)	235 (28.4)
Friends	104 (26.4)	83 (19.2)	83 (19.3)	10 (30.3)	187 (22.6)
Religious group	88 (22.3)	98 (22.7)	101 (23.4)	10 (30.3)	186 (22.5)
Professional group	28 (7.2)	18 (4.2)	25 (5.8)	4 (12.1)	46 (5.5)
Social group	35 (8.8	21 (4.8)	27(6.3)	3 (9.1)	56 (6.8)
Community	23 (5.9)	12 (2.8)	18(4.2)	2(6.1)	35 (4.2)
Philanthropists	6 (1.5)	3 (0.7)	3(0.7)	0 (0.0)	9 (1.1)
Politicians	12 (3.1)	10 (2.3)	10(2.3)	0 (0.0)	22 (2.7)
Governments	4 (1.0)	5 (1.2)	6(1.4)	0 (0.0)	9 (1.1)
NGO	1 (0.3)	3 (0.7)	2(0.5)	0 (0.0)	4 (0.5)

percentages are in parenthesis

^a^ computed for older adults with a living spouse only

^b^ computed for older adults with a living child only

^c^ unmarried include the widows, single, divorced, and separated

### Complementary support scores of older adults in Oyo State

The complementary support scores indicate the extent of older adults’ access to complementary support. Of the total obtainable score (100), the overall median complementary support score was about 30% (interquartile range [IQR] = 24), with only a quarter of the respondents scoring above 42%, regardless of gender (Figure not shown). However, there were noticeable gender disparities in some background characteristics of the respondents.

**Fig. 1:**
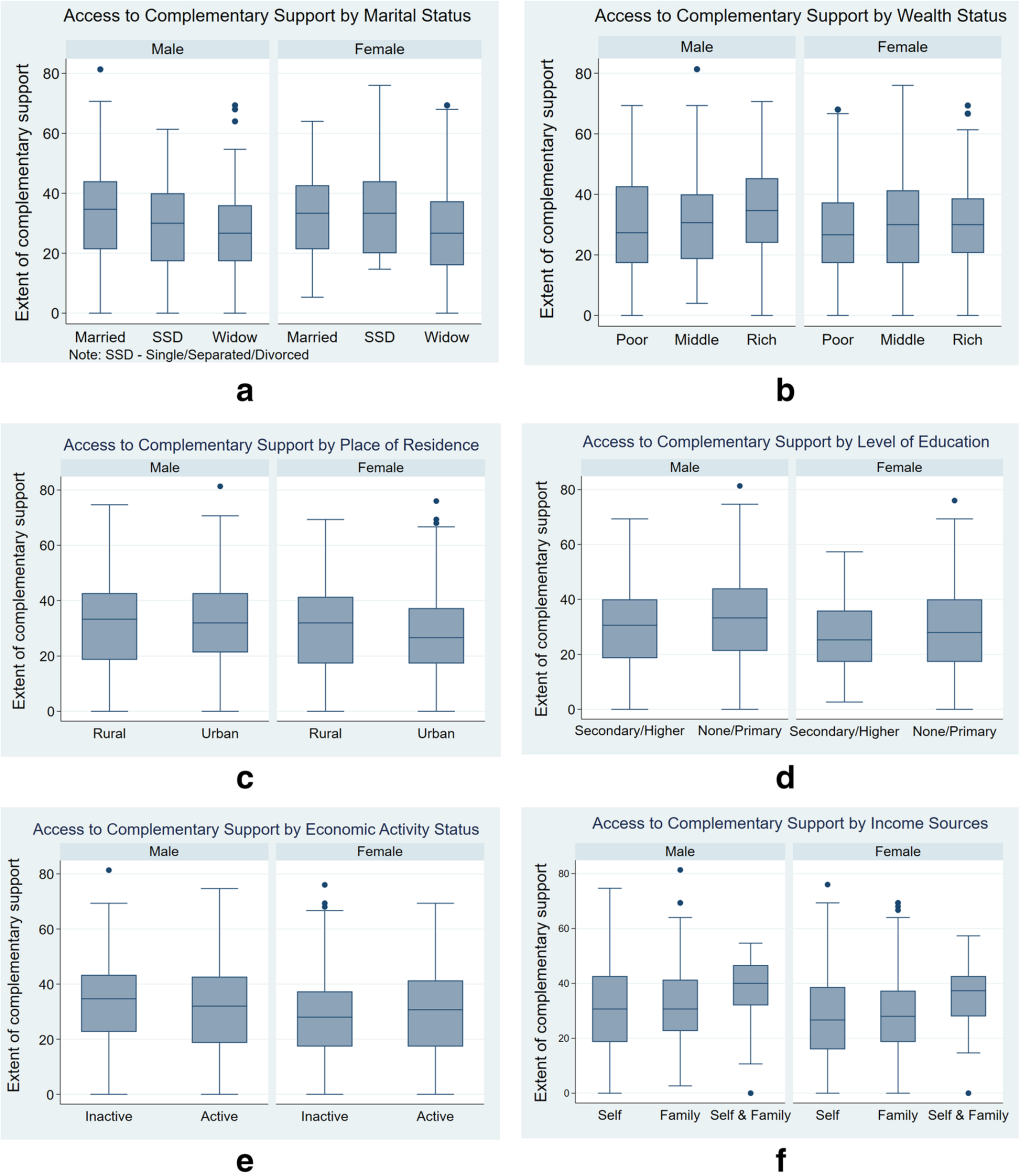
Pattern of complementary support by gender and selected background characteristics

Figure 1 (panels a-f) presents the patterns of complementary support by gender and some background characteristics. As shown in Fig. 1a, the median score, by marital status, was lowest (median = 26%; IQR = 18) for the widow(er)s with a quarter of the respondents scoring 36% and above, compared to the currently married with the highest median score (median = 36%; IQR = 23) and one-quarter scoring 44% and above. This disparity was similar across both genders (Fig. 1a). Also, older men in the lower wealth status had the least median score (median = 28%; IQR = 24) of complementary support (with three-quarters of them having 42% or below) while the rich had the highest median score (median = 36%; IQR = 22) with half of them scoring above the median score (Fig. 1b). This pattern is similar to that of their women counterparts, though the 75^th^ percentile score of the women in poor and rich wealth statuses was lower (36% versus 38%) compared to men (42% versus 45%). The distribution of complementary support scores was similar across the rural–urban residences, though urban women had a slightly lower median score (median = 28%; IQR = 20) compared to their rural counterparts (median = 33%; IQR = 24) (Fig. 1c). Education also presented similar distribution, except that women with secondary/higher education had a lower median score (median = 26%; IQR = 18) with about half scoring below the median unlike men, half of whom scored above 32% (IQR = 20) (Fig. 1d).

The economic activity of older adults, as shown in Fig. 1e, did not present any significant disparity except between the male and female genders. While economically inactive women had a complementary support median score of 28% (IQR = 18), their men counterparts had a complementary support median score of 34% (IQR = 20). In Fig. 1f, some disparities in access to complementary support scores were observed between the men and women, especially with respect to their sources of income. Those who depended on themselves and family for income had the highest complementary support score compared to those who depended on themselves for income, though the score is slightly lower among women (median = 37%; IQR = 14) compared to men (median = 40%; IQR = 12) with about half having below the median score in each group.

### Factors associated with the extent of access to complementary support in later life

The results in Table 3 show the factors associated with the access to complementary support by older men and women in the study area. The multiple regression estimates (β) indicated that, overall, access to complementary support was associated with the age, level of education, marital status, religious affiliation, wealth status, and sources of income of the respondents (*p* < 0.05). However, the gender analyses show that of the identified factors in the overall data above, age, education, and wealth status were not significant predictors of older women’s access to complementary support. Also, for men, though wealth status was not a predictor of their access to complementary support, economic activity status was key.

**Table 3:** Multiple regression analysis of the factors associated with access to complementary support among older adults in Oyo State, Nigeria

Socio-demographic Characteristics	Overall		Male		Female	
	β (95% C. I.)	SE	β (95% C. I.)	SE	β (95% C. I.)	SE
**Age group**						
< 70 ^R^	-	-				
70 – 79	0.778 (-1.657 – 3.213)	1.241	-0.198 (-3.855 – 3.458)	1.860	0.680 (-2.663 – 4.023)	1.701
80 or older	-3.649 (-6.460 – -0.838)*	1.432	-6.745 (-11.115 – -2.375)**	2.222	-2.463 (-6.260 – 1.333)	1.932
**Place of residence**						
Rural ^R^						
Urban	-0.839 (-2.929 – 1.251)	1.065	-0.446 (-3.579 – 2.687)	1.593	-1.360 (-4.209 – 1.489)	1.449
**Level of education**						
Secondary/higher ^R^						
Primary/None	4.365 (1.511 – 7.218)**	1.454	5.371 (1.685 – 9.057)**	1.875	4.048 (-0.681 – 8.776)	2.405
**Marital Status**						
Currently married ^R^						
Never married/separated/divorced	-2.228 (-6.066 – 1.609)	1.955	-5.554 (-10.433 – -0.675)*	2.481	3.877 (-2.694 – 10.447)	3.343
Widow	-6.285 (-8.556 – -4.015)***	1.157	-5.677 (-9.756 – -1.599)**	2.074	-5.419 (-8.654 – -2.184)**	1.646
**Religion**						
Christianity ^R^						
Others (Islam & Traditional)	5.100 (3.000 – 7.200)***	1.070	3.882 (0.773 – 6.990)*	1.581	6.067 (3.180 – 8.954)***	1.469
**Wealth status**						
Poor ^R^						
Middle	0.648 (-1.830 – 3.125)	1.262	-0.209 (-4.145 – 3.726)	2.001	1.555 (-1.680 – 4.790)	1.646
Rich	3.315 (0.667 – 5.963)*	1.349	3.872 (-0.130 – 7.873)	2.035	2.259 (-1.430 – 5.949)	1.877
**Occupational Status**						
Not working ^R^						
Working	-1.614 (-4.321 – 1.092)	1.379	-4.789 (-8.729 – -0.850)*	2.003	1.445 (-2.335 – 5.225)	1.923
**Main sources of income**						
Self						
Children/family	1.972 (-0.834 – 4.778)	1.430	-0.081 (-4.379 – 4.218)	2.186	4.205 (0.416 – 7.993)*	1.927
Both self and children/family	5.510 (1.710 – 9.309)**	1.935	4.988 (-0.369 – 10.345)	2.724	6.457 (1.016 – 11.898)*	2.768

^*SD* Standard deviation; β adjusted regression coefficient, *C.I*. Confidence Interval, *SE* Standard Error, *R* Reference category^

^*p<0.05^

^**p<0.01^

^***p<0.001^

Specifically, older adults in the oldest age group 80 years or older (β = -3.65, *p* < 0.05, 95% C.I. = -6.460 – -0.838) had significantly reduced access to complementary support by 3.65 compared to their younger counterparts below 70 years, holding all other factors constant. This result is consistent for older men only in the gender analyses at *p* < 0.01. Similarly, being a widow (β = -6.285, *p* < 0.001, 95% C.I. = -8.556 – -4.015) results in reduced access to complementary support by 6.285 compared to being currently married, holding all other factors constant. The results were similar for both genders.

Conversely, some older adults’ characteristics predisposed them to increased access to complementary support. As shown in the table, in the overall data, being in Islamic and Traditional religious groups (β = 5.100, *p* < 0.001; 95% C.I. = 3.000 – 7.200) significantly increased older adults’ access to complementary support by 5.100 relative to being a Christian, other factors remaining constant. This finding was consistently similar for both older men and women in the study area. Also, having below secondary education (β = 4.365, *p* < 0.01, 95% C.I. = 1.511 – 7.218) yields an increase in access to complementary support among older adults by 4.365 compared to having secondary or higher education, all other factors remaining constant. However, this result was consistent only in the men’s data. The analysis of the overall data further revealed that being in the rich wealth status yields increased access to complementary support (β = 3.315, *p* < 0.05; 95% C.I. = 0.667 – 5.963) by 3.315 holding other factors constant. The result was not, however, significant when analysed by gender. Also, older adults whose main sources of income comprise both self and family had a significant increase (β = 5.510, *p* < 0.01, 95% C.I. = 1.710 – 9.309) in access to complementary support by 5.510 compared to their counterparts who were self-dependent. However, this result was only consistent for older women. Though economic activity showed no significant association in the overall data, it was a significant predictor of men’s access to complementary support; being economically active results in reduced access to complementary support (β = 4.789, *p* < 0.05, 95% C.I. = -8.729 – -0.850) by 4.789, other factors held constant.

## Discussion

Access to complementary support plays important role in the life of older adults who face an increased risk of vulnerability to health impairment and dependency on care as they advance in age. It does not only contribute to the healthy living of older adults, but it also contributes to global success in realizing the sustainable development goals target on ensuring health and wellbeing for people of all ages (goal 3). This study investigated the extent of access to complementary supports and its associated factors among older adults in Oyo State, South-Western Nigeria. For this study, the sources of support that are expected to complement one another, irrespective of the type of support, include immediate family (children and spouse), extended family, and non-family networks.

The findings of this study revealed that children are the major caregivers of older adults in Oyo State, as reported by 85% of the respondents who claimed to have received substantial support from the children in the past 12 months. This is in tandem with the findings of Osamor (2015) who stated that 93% of older adults in Ibadan reported receipt of social support from immediate family members, especially children [24]. The few who received little or no support from the children may be linked to loss of children and children’s financial incapacity as identified by existing studies [9, 10, 36, 37]. Meanwhile, it is worthy of note that both older men and women had similar access to support from children, and by extension, from other immediate and extended family members, contrary to the findings of Katz and colleagues [26] and Noël-Miller [27] who argued that children were more likely to support their mother than the father [26, 27].

Though spouses have also been identified as primary caregivers in later life [10, 24, 27], our finding has revealed some exceptions – a high prevalence of support from the spouse is achievable only among those with living spouses. For instance, in our study, of the respondents who have a living spouse, about 76% received spousal support which is lacking for the widows, single, divorced, and the separated. This finding agrees with other studies which reported that a large proportion of older adults with living spouses receive support from these spouses [38].

However, having a living spouse does not guarantee access to spousal support. This is because spousal support may be limited or completely unavailable due to financial incapacity and health impairment [39]. In reality, spousal support has often been threatened by a high prevalence of widowhood, separation, and divorce among older people [39]. Of the total sample of our study, only 48% reported having received spousal support in the last 12 months while 45% never received any support (table not shown). The high proportion of widowhood, separation, divorce, and unmarried (52%) might have accounted for the low spousal support when estimated based on the total sample. The effect could be more pronounced among women with a higher proportion of widowhood (65%) compared to men, 18%. Also, though more respondents claimed to be supported by children, it did not necessarily imply that less support comes from the spouse. As observed by Blomgren and colleagues in 2008, children most often do not live with their aged parents, hence, their support is not as intensive and frequent as the support from spouses who live with their partner and often provides domestic and emotional support [38].

In addition to the support received from children and spouses, older adults also accessed support from other sources, though to a lesser degree. For instance, an appreciable proportion of the respondents in our study received much support from other sources including siblings, in-laws, and neighbours (Table 2). This finding is similar to that of Osamor (2015) who stated that (55%) of older adults were able to access support from non-family members, especially friends [24]. However, our study demonstrates higher access to extended family support compared to Okumagba’s (2011) study which reported that only a quarter of older adults in South-Southern Nigeria received support from extended family [40]. This suggests cultural variations in access to support from extended family. Nevertheless, in most cases, support expected from non-family members is not as much as those expected from family members. Wu and Pollard (1998) also supported the claim, stating that only 15% of older adults did access support from friends [14]. This is because family support is often driven by a sense of reciprocity and devotedness to the recipient while non-family support is driven by a sense of solidarity, societal norms, and personal relationship with the recipient [41].

Access to non-family support is not only dependent on proximity to the benefactor, except neighbours, it is also dependent on the closeness and frequency of contact with the supporter, the value attached to their relationship, and the extent of benefactors’ ability to bear the burden of providing support. For instance, while neighbours are able to provide support for emergency needs due to geographical proximity, friends and religious groups may provide emotional support amidst loneliness, anxiety, or depression through social interaction and encouragement from religious perspectives [42].

Access to complementary support was associated with older adults’ level of education, marital status, religious affiliation, wealth status, and income sources [20, 22]. Though higher education has often been found as a strong factor that stems from many social, behavioural, and public health problems, our study noted a contrary relationship. A low level of education is associated with increased access to complementary support. This is similar to the finding of other authors in other contexts [22] and Nigeria [24, 43, 44]. This outcome may be linked to the role of education as a major driver of migration for older adults’ children who should be available to provide support and also attract external support for their parents. Educated elderlies who were plausibly able to afford the cost of their children’s education were likely to be more affected by the children’s migration, hence, their lower access to complementary support. Besides, education is also theorised to play a role in modernisation’s ideology of the nuclear family system [36]. However, more educated, and thus likely wealthier, older adults might be able to pay for private care/support or receive financial remittance from their adult children.

Also, widow(er)s in Oyo State had reduced access to complementary support by 6.285 compared to the married. This is because older people who are married are able to access more support either from their spouse directly or from the spouse’s social network. This is consistent with Osamor’s (2015) finding which associated older adults’ receipt of support from their family with being married [24]. Our finding, however, contrasts the finding of Barrett and Lynch (1999) who stated that widowed and unmarried older Canadians have larger support networks to help than the married [21], and that of Wu and Pollard (1998) who reported no association between marital status and receipt of social support in Canada [14]. Though the finding of our study may be an update to the existing evidence, the contrast may be linked to the different social and geographical settings of our study compared to others.

With respect to religion, our finding indicated that increased access to complementary support is associated with non-Christian religion. This aligns with Osamor’s finding in 2015 [24]. This relationship may be linked to the practice of polygyny, having a large family size, and living in clusters which are more prevalent among Muslims and traditionalists compared to the Christians. Existing studies have also argued that older adults with large household sizes or living with others have greater access to support compared to the otherwise groups [45, 46].

Moreover, the study further found that older adults’ main sources of income play important role in their access to complementary support. Those who depend on themselves and their children for income were more likely to have increased access to complementary support from children and through their network as well as the children’s network. Though the association faded in the male data, it is key and more significant among older women.

### Strengths and limitations

One of the major strengths of this study is its quantitative approach which was based on a representative sample of the study area. The study, however, has some limitations. The finding of this study is based on quantitative and cross-sectional data, hence, causality could not be inferred in any way. Also, the study was based on the analysis of self-reported data of the respondents without any mechanism to validate the reported responses. Further, older adults with chronic disease and dementia were excluded from this study, even though they were most likely to receive higher degrees of support.

## Conclusion

This study concludes that access to complementary support was low among older adults. Though a sizeable proportion of them received support from other sources to complement spouses’ and children’s support, fewer proportion received support from other sources, especially the non-family sources. This suggests that the non-family sources may not be dependable for major or long-term needs. Access to complementary support to a great extent depends on the older adult’s age, marital status, educational status, religious affiliation, and main sources of income. Given these, widowed and childless older adults are likely to experience a greater burden of lack of support. Further commitment of the government, non-governmental organisations, religious and social groups, and community members to the welfare of older adults are recommended. Future research should complement the quantitative evidence with qualitative data for better insight into the quality and nature of complementary supports. A study inclusive of older people with dementia and chronic disease is also needed to investigate how their support networks differ. Finally, considering the dynamics of needs in later life, a longitudinal study is needed to investigate how the support network and experience of older adults change over time.

## Data Availability

The data supporting the findings of this study are available on request from the corresponding author, JWM. The data are not publicly available due to their containing information that could compromise the privacy of research participants.

## Abbreviations

EA: Enumeration Area
LGA: Local Government Area
NBS: National Bureau of Statistics
PSU: Primary Sampling Unit
REDCap: Research Electronic Data Capture
SSA: Sub-Saharan Africa
